# A scoping review of symptom clusters in adult leukemia patients undergoing chemotherapy

**DOI:** 10.1097/MD.0000000000045165

**Published:** 2025-10-31

**Authors:** Shujia Liu, Lei Wang, Jiating Wang, Xiaodong Xu, Kun Zhang, Ting Wang, Xia Yan

**Affiliations:** a Department of Hematology, Peking University People’s Hospital, Peking University Institute of Hematology, National Clinical Research Center for Hematologic Disease, Beijing, China.

**Keywords:** adult leukemia patients, leukemia, scoping review, symptom clusters during chemotherapy, symptom management

## Abstract

**Background:**

Patients with leukemia undergoing chemotherapy frequently present with a cluster of co-occurring symptoms, which exacerbate both physical and mental burdens and may worsen prognosis. A comprehensive review of the types, characteristics, and research status of these symptom clusters is thus necessary to inform and improve symptom management strategies.

**Methods:**

A systematic search was conducted in 8 databases, including PubMed, Embase, CINAHL, Web of Science, CNKI, Wanfang Database, VIP Database, and China Biomedical Literature Database, for relevant studies on symptom clusters in leukemia patients during chemotherapy. The retrieval period spanned from the inception of the databases until June 28, 2024.

**Results:**

Nine studies identified 11 symptom clusters concluding skin-related, oral-related, self-image impairment, gastrointestinal, nervous system-related, psychological, energy deficiency, adverse reaction, pain-related, nutritional, and disease behavior symptom clusters. Notably, the gastrointestinal and psychological symptom clusters were the most prevalent.

**Conclusion:**

Leukemia patients undergoing chemotherapy commonly experience multiple symptom clusters, yet there are substantial variations in the internal composition of these clusters. Future research should focus on exploring specific symptom assessment tools for leukemia patients and integrating multiple advanced analysis methods, such as machine learning, to improve the accuracy of symptom cluster identification and support the formulation of targeted management strategies.

## 1. Introduction

Leukemia, a clonal proliferative disorder of hematopoietic stem cells, ranks 15th in global cancer incidence and is the most common hematopoietic malignancy.^[1]^ A Chinese study that reviewed leukemia incidence and mortality data from the 2005-2007 China Cancer Registry Annual Report indicated an increasing trend in age-standardized incidence since 2005.^[2]^ Another study, which collected and analyzed the epidemiological characteristics and incidence trends of leukemia in a specific Chinese city from 1972 to 2021, also demonstrated an increasing incidence over the past five decades.^[3]^ The latest data from the Domestic Cancer Registry Center released in 2022 showed an incidence rate of 5.0/100,000 and a mortality rate of 3.425/100,000, with both rates continuing to rise.^[4]^ Clearly, leukemia has emerged as a major malignant tumor threatening the health of Chinese residents and warrants the close attention of medical professionals.

Immunotherapy, including monoclonal antibodies such as blinatumomab and inotuzumab ozogamicin, and chimeric antigen receptor T-cell therapies such as tisagenlecleucel and brexucabtagene autoleucel, has shown promising results in relapsed or refractory B-cell Acute Lymphoblastic Leukemia (B-ALL), with notable remission rates and manageable side effects. Bone marrow transplantation, particularly allogeneic stem cell transplantation, offers potential cures for high-risk or relapsed cases but poses risks such as graft-versus-host disease and infections. Chemotherapy remains the primary treatment, employing multi-phase regimens tailored to individual risk profiles. Despite these advancements, treatment resistance, toxicity, and accessibility issues remain.^[5]^ Leukemia patients often experience multiple symptoms, such as nausea, vomiting,^[6]^ fever,^[7]^ decreased muscle strength, and fatigue,^[8]^ due to both the disease and chemotherapy. Compared with a single symptom, multiple symptoms may have synergistic and exacerbating effects. The consequences are not merely the sum of the effects of individual symptoms but show a multiplicative trend, significantly impacting patient prognosis and reducing quality of life.^[9,10]^

Symptom clusters, first introduced by Dodd in 2001,^[11]^ refer to two or more simultaneously occurring and interrelated symptoms.^[12]^ The management of symptom clusters can effectively improve the precision and efficiency of symptom management.^[13]^ Effective management of symptom clusters may reduce treatment discontinuation and improve survival outcomes in leukemia patients. The study of symptom clusters is crucial for the early identification and effective management of symptoms in leukemia patients during chemotherapy. At present, some scholars have explored the symptom clusters of leukemia patients.^[14,15]^ However, the analysis methods, types, and characteristics of symptom clusters in various studies are not clear and there is great heterogeneity, which brings obstacles to the management of symptom clusters. Therefore, this scoping review endeavors to examine the identification methods, types, characteristics, and variation patterns of symptom clusters in different chemotherapy stages among adult leukemia patients both domestically and internationally. By doing so, it aims to identify the existing problems in current research on leukemia patient symptom clusters and clarify future research directions, thereby promoting the development of symptom cluster management in leukemia patients and improving the efficiency of symptom management.

## 2. Materials and methods

This research report adhered to the Preferred Reporting Items for Systematic reviews and Meta-Analyses extension for Scoping Reviews (PRISMA-ScR) guidelines and was based on the scoping review report framework proposed by the Joanna Briggs Institute (JBI).^[16]^ A comprehensive scoping review was conducted on the assessment tools for symptoms and the types and characteristics of symptom clusters in leukemia patients during chemotherapy. The objective was to establish a foundation for further optimizing the management of symptom clusters in leukemia patients during chemotherapy. This study is a scoping review of previously published literature. As no original data were collected from human or animal subjects, ethical approval was not required for this work.

### 2.1. Defining the research questions

The research questions of this scoping review were clearly defined as follows: What are the assessment tools for symptom clusters in leukemia patients during chemotherapy? What are the types of symptom clusters in leukemia patients during chemotherapy? Is the internal composition of symptom clusters stable?

### 2.2. Inclusion and exclusion criteria for literature

Inclusion criteria were determined based on the participants (P), concept (C), and context (C). Research participants: leukemia patients aged ≥ 18 years. Concept: involving the identification of symptom clusters or the simultaneous existence and correlation of multiple symptoms. Context: patients receiving chemotherapy in outpatient or inpatient settings. Exclusion criteria: non-English and Chinese literature, literature whose full text and research protocol cannot be obtained, conferences, reviews, and opinions.

### 2.3. Search strategy

The databases of PubMed, Embase, CINAHL, Web of Science, CNKI, Wanfang Database, VIP Database, and China Biomedical Literature Database were searched. The retrieval time limit was from the establishment of the database to June 28, 2024. The English search terms are “Leukemia/leukaemia*/leukemia*/leukemic/leukosis,” “syndrome/symptom cluster*/symptom constellation*/concurrent symptom*/multiple symptom*/symptom combination.” Chinese search terms “leukemia,” “symptom cluster/symptom set/symptom cluster.” A combination of subject headings and keywords was used to search and references were tracked. The search strategies for PubMed and Web of Science were as follows:

Pubmed ((((((syndrome[MeSH Terms]) OR (symptom cluster*[Title/Abstract])) OR (symptom constellation*[Title/Abstract])) OR (concurrent symptom*[Title/Abstract])) OR (multiple symptom*[Title/Abstract])) OR (symptom combination[Title/Abstract])) AND (((leukemic[Title/Abstract]) OR (leukosis[Title/Abstract])) OR (((leukaemia*[Title/Abstract]) OR (leukemia*[Title/Abstract])) OR (Leukemia[MeSH Terms]))).

Web of science ((((TS = (leukemic)) OR TS = (leukosis)) OR TS = (leukaemia*)) OR TS = (leukemia*)) AND (((((TS = (symptom cluster*)) OR TS = (symptom constellation*)) OR TS = (concurrent symptom*)) OR TS = (multiple symptom*)) OR TS = (symptom combination))

### 2.4. Literature screening and data extraction and analysis

The retrieved studies were imported into Endnote software for deduplication. Subsequently, two researchers with experience in the hematology department and training in evidence-based scientific research independently screened the literature by reading the titles and abstracts in accordance with the inclusion and exclusion criteria. Subsequently, the full texts were read for further screening. In case of any questions or discrepancies, a discussion was held with a third researcher to finalize the included studies. The information extracted from the included studies encompassed authors, countries, research types, sample sizes, treatment stages, assessment tools, usage dimensions, analysis methods, and symptom clusters, which was then summarized and analyzed.

### 2.5. Literature quality assessment

The quality of the included studies was assessed based on their study design, using the corresponding JBI critical appraisal tools: the JBI Critical Appraisal Checklist for Analytical Cross-Sectional Studies was applied for cross-sectional studies, and the JBI Critical Appraisal Checklist for Cohort Studies was applied for cohort studies. Studies were graded as: A (fully met all criteria), B (partially met criteria), or C (did not meet any criteria). Only studies rated as Grade A or B were ultimately included in the analysis.

## 3. Results

### 3.1. Literature screening results

**Figure 1.**
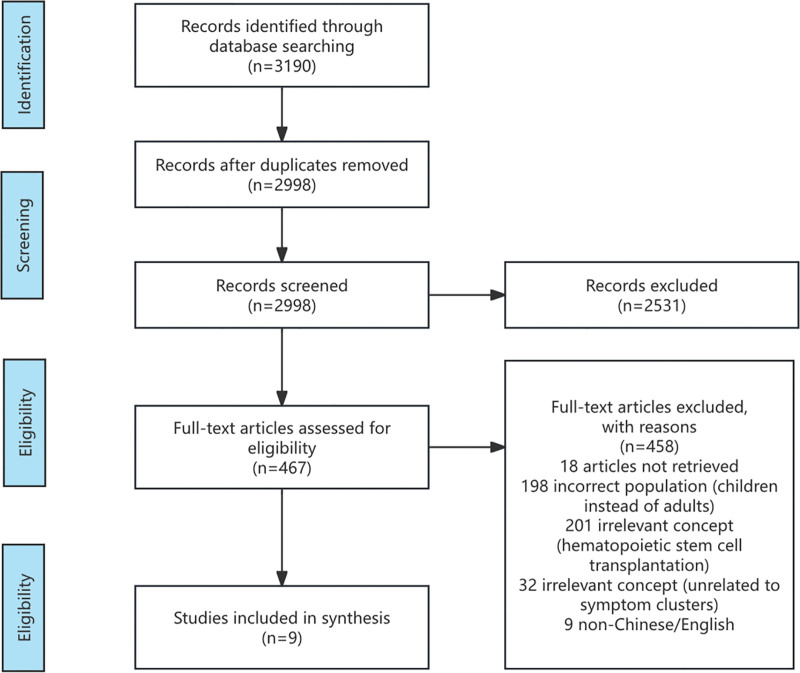
PRISMA-ScR flow diagram illustrating the process of study identification, screening, eligibility assessment, and inclusion in the scoping review. (A total of 3190 records were initially identified from eight databases. After removing duplicates, 2998 records were screened by title and abstract. Following a full-text assessment for eligibility, 9 studies were ultimately included in the review.) PRISMA-ScR = preferred reporting items for systematic reviews and meta-analyses extension for scoping reviews.

A total of 3190 studies were initially retrieved, including 1400 from PubMed, 1554 from Web of science, 46 from CINHAL, 76 from Embase, 29 from CNKI, 21 from China Biomedical Literature Database, 16 from VIP, and 44 from Wanfang. After removing duplicate studies, 2998 studies remained. Further title and abstract screening, identification and inclusion of studies are documented in the PRISMA-ScR flow diagram (Fig. 1), yielding 9 final papers for the review. The results of the quality assessment were all rated as Grade A or B. The literature information is shown in Table 1.

**Table 1:** Characteristics of included literature.

Author/year	Country	Study design	Sample size	Tools used	Symptom cluster types	Quality assessment
Xixi Yin (2020)^[15]^	China	Cross Sectional Study	198	MSAS-Ch	Oral-related	A
					Gastrointestinal	
					Psychological	
					Energy deficiency	
					Pain	
Ting Zhang (2020)^[17]^	China	Cross Sectional Study	104	MSAS-Ch	Self-image impairment	B
					Gastrointestinal	
					Nerve	
					Psychological	
					Adverse reaction	
Ting Zhang (2020)^[18]^	China	Longitudinal study	116	MSAS-Ch	Skin	B
					Oral-related	
					Self-image impairment	
					Gastrointestinal	
					Nerve	
					Psychological	
					Energy deficiency	
					Adverse reaction	
					Pain	
					Nutrition	
Guifang Yang (2021)^[19]^	China	Longitudinal study	130	MSAS-Ch	Self-image impairment	B
					Gastrointestinal	
					Nerve	
					Psychological	
					Energy deficiency	
					Pain	
					Nutrition	
Cherwin CH (2017)^[20]^	USA	Longitudinal study	105	MSAS, MDASI	Self-image impairment	A
					Gastrointestinal	
					Nerve	
					Psychological	
					Energy deficiency	
Fengjiao Chen (2021)^[21]^	China	Cross Sectional Study	132	MSAS-Ch	Gastrointestinal	A
					Psychological	
					Nutrition	
					Illness behavior	
Changlan Liang (2017)^[22]^	China	Cross Sectional Study	191	MDASI	Gastrointestinal	B
					Nerve	
					Psychological	
					Energy deficiency	
Choi Wan Chan (2020)^[23]^	Hong Kong, China	Cross Sectional Study	64	MDASI	Gastrointestinal	B
					Nerve	
					Illness behavior	
Juanjuan Li (2022)^[24]^	China	Cross Sectional Study	86	Self-designed questionnaire	Skin	A
					Oral-related	
					Self-image impairment	
					Gastrointestinal	
					Nerve	
					Psychological	

The quality of the included studies was assessed based on their study design, using the corresponding JBI critical appraisal tools: the JBI Critical Appraisal Checklist for Analytical Cross-Sectional Studies was applied for cross-sectional studies, and the JBI Critical Appraisal Checklist for Cohort Studies was applied for cohort studies. Studies were graded as: A (fully met all criteria), B (partially met criteria), or C (did not meet any criteria). Only studies rated as grade A or B were ultimately included in the analysis. MSAS = memorial symptom assessment scale, MSAS-Ch = memorial symptom assessment scale-Chinese version, MDASI = M.D. Anderson Symptom Inventory.

### 3.2. Assessment tools for symptom clusters in leukemia patients

Appropriate assessment tools are of great significance for symptom assessment, symptom cluster extraction, correlation factor analysis, and intervention method formulation. The nine included studies employed three distinct assessment tools: the memorial symptom assessment scale (MSAS), the M.D. Anderson Symptom Inventory (MDASI), and self-designed questionnaires. Among these, the MSAS was the most frequently employed tool, being utilized in six studies.^[15,17–21]^ The MDASI was utilized in three studies.^[20,22,23]^ One study utilized a self-designed scale,^[24]^ and one study applied both MSAS and MDASI.^[20]^ Unifying the assessment tools for symptom clusters and determining the best identification method will help to verify symptom clusters and assist in implementing symptom management strategies in clinical practice. All 9 included studies in this article used factor analysis methods to identify symptom clusters.

### 3.3. Types and characteristics of symptom clusters in leukemia patients

A total of 11 symptom clusters were extracted from the 9 included studies, primarily involving two dimensions: physiological and psychological. These included skin-related symptom cluster, oral-related symptom cluster, self-image impairment symptom cluster, gastrointestinal symptom cluster, nervous system-related symptom cluster, psychological symptom cluster, energy deficiency symptom cluster, adverse reaction symptom cluster, pain-related symptom cluster, nutritional symptom cluster, and illness behavior symptom cluster. Among them, the gastrointestinal symptom cluster and the psychological symptom cluster were the most common and relatively stable in internal composition during chemotherapy in leukemia patients. These two symptom clusters were identified in all 9 studies. The gastrointestinal symptom cluster typically comprised nausea, vomiting, decreased appetite, constipation, etc; the psychological symptom cluster mainly included tension, worry, anxiety, sadness, etc. The next most common were the nervous system-related symptom cluster, the energy deficiency symptom cluster, and the self-image impairment symptom cluster. Most of the included studies in this study focused on leukemia patients during chemotherapy. The symptom clusters of patients in different courses exhibited dynamic changes, with diverse symptom clusters and significant differences in the composition of most symptom clusters. However, the gastrointestinal-related symptom cluster and the psychological symptom cluster persisted throughout all stages.

## 4. Discussion

### 4.1. Diverse symptom clusters during chemotherapy in leukemia patients, with most cluster compositions remaining unclear

Leukemia patients frequently experience multiple symptom clusters during chemotherapy. Current studies report approximately 11 types of symptom clusters, including gastrointestinal symptom clusters, psychological symptom clusters, and neurological symptom clusters. Among these, gastrointestinal and psychological symptom clusters are the most prevalent and exhibit internal stability throughout the course of chemotherapy, as consistently documented in all nine studies included in this review. In contrast, other symptom clusters—demonstrate significant compositional variations and inconsistent nomenclature across different studies. This variability underscores the necessity for standardization and further investigation of these symptoms.

Gastrointestinal symptom clusters consistently include nausea and vomiting as core symptoms across the studies reviewed, some also encompass additional symptoms such as constipation and anorexia. Variations exist in their nomenclature, with terms like “vomiting symptom cluster” or “gastrointestinal symptom cluster” being utilized. Chemotherapy, the primary treatment for leukemia, targets cancer cells but induces a range of toxic side effects, among which nausea and vomiting are the most frequently reported adverse reactions.^[25]^ Nausea and vomiting often co-occur and influence one another (for instance, vomiting may exacerbate nausea, creating a vicious cycle); consequently, clinical interventions typically involve the prophylactic administration of antiemetics. However, these antiemetics can themselves lead to side effects such as constipation.^[26]^ Additionally, factors such as reduced physical activity and decreased oral intake during chemotherapy further elevate the risk of constipation. Studies indicate that the incidence of constipation among leukemia patients undergoing chemotherapy ranges from 50% to 80%.^[26]^ The concurrent manifestation of these symptoms suggests that gastrointestinal symptom clusters represent not merely a simple aggregation of individual symptoms but rather an interplay of multiple contributing factors. Collectively, these symptom clusters may significantly impact the effective implementation of chemotherapy and patient prognosis through adverse effects on nutritional intake, treatment tolerance, and adherence to therapy.

All included studies consistently identified tension, worry, and sadness as core manifestations of psychological symptom clusters. For leukemia patients confronting this profound negative life event, the disease precipitates not only abrupt physiological decline but also sudden social role disruption (e.g., suspended occupational/familial responsibilities) and profound trepidation regarding protracted treatment regimens.^[27]^ Treatment-associated distress, illness uncertainty, and chemotherapy-induced adverse effects (e.g., fatigue, nausea) further compound psychological burdens. These synergistic mechanisms thus position psychological symptom clusters as prevalent and clinically consequential entities during chemotherapy.

The pervasive coexistence of gastrointestinal and psychological symptom clusters underscores their critical role as determinants of patients’ global status during chemotherapy, directly impairing quality of life while establishing mutually reinforcing loops through bidirectional interactions—where anxiety exacerbates gastrointestinal distress, which subsequently amplifies psychological burden—ultimately compromising treatment adherence and therapeutic efficacy. Crucially, symptom clusters transcend mere symptom aggregates, functioning as pathophysiologically interconnected constellations whose synergistic impact exceeds the sum of individual effects; this approach comprehensively captures chemotherapy’s multisystemic toxicity, exemplified by gastrointestinal clusters (nausea/vomiting/constipation) and psychological clusters (anxiety/worry) co-occurring through mind-body interactions (e.g., nausea-induced anxiety further intensifying nausea)—complex linkages unobservable via isolated symptom analysis. Importantly, clusters directly govern clinical outcomes by impairing nutritional intake (gastrointestinal clusters), reducing treatment adherence (psychological clusters), and delaying functional recovery, collectively diminishing therapeutic response—a multidimensional burden resistant to single-symptom interventions. Clinically, clusters serve as precision therapeutic targets: prioritizing core symptom alleviation (e.g., antiemetics for nausea) demonstrates superior efficiency versus fragmented management. Consequently, our cluster-centric paradigm elucidates holistic chemotherapy-induced symptom patterns, providing an empirical foundation for integrated management strategies.

Current research on symptom clusters remains nascent. Beyond gastrointestinal and psychological clusters, significant compositional heterogeneity exists in other prevalent clusters such as neurological and energy deficiency clusters. For neurological clusters, manifestations vary substantially across studies—encompassing peripheral neuropathy (limb numbness), xerostomia, and cognitive impairment;^[18]^ or somnolence, dry mouth, and dizziness.^[17]^ Some studies isolated distinct “cephalic-related clusters” (drowsiness, headache, vertigo)^,[24]^ while others consolidated neurological and psychological symptoms into “neuropsychological clusters.” Similarly, energy deficiency clusters predominantly feature fatigue, dyspnea, somnolence, concentration difficulties, and lack of energy,^[22]^ though are variably termed “fatigue clusters” in certain literature.^[19]^ This inconsistency in nomenclature and cluster constitution underscores the absence of standardized conceptual frameworks for core symptom cluster phenotypes.

Critical knowledge gaps persist in symptom cluster research, beginning with unelucidated pathophysiological mechanisms—specifically how chemotherapeutic agents induce multisystem symptoms and establish inter-symptom associations within clusters. Furthermore, ambiguous core definitions plague the field, particularly regarding undetermined thresholds for cluster composition size and symptom association strength, driving inconsistent core symptom selection and clustering methodologies. Most critically, nomenclatural and constitutive disarray manifests as divergent labeling of phenotypically similar clusters (e.g., “vomiting cluster” versus “gastrointestinal cluster”) and variable consolidation/fragmentation rules across studies (notably the inconsistent handling of neuropsychological clusters), severely compromising cross-study comparability and clinical translatability. These interconnected deficiencies fundamentally constrain accurate assessment of cluster-pathobiology relationships while impeding the translation of clusters into precision therapeutic targets. Consequently, future research must establish standardized core definitions through integrated pathophysiological-statistical frameworks, determine cluster stability patterns across treatment phases, and ultimately enable personalized symptom management strategies that improve patient outcomes and quality of life.

### 4.2. There are various symptom assessment tools for leukemia patients, and specific symptom cluster assessment tools need to be further explored

Appropriate assessment tools play a significant role in symptom evaluation, symptom cluster extraction, analysis of related factors, and the development of intervention strategies. Different assessment tools may lead to significant differences in the types, numbers, and internal composition of symptom clusters. In the research on symptom clusters during chemotherapy in leukemia patients, the identification of symptom clusters involves not only symptom assessment but also the choice of analytical methods. Standardizing the assessment tools for symptom clusters and determining the best identification methods will help verify the existence of symptom clusters and provide more reliable symptom management strategies for clinical practice. Otherwise, the generalizability and cross-study applicability of research findings will be significantly limited. Assessment tools specifically designed and validated for particular patient population are more specific because they can accurately identify symptoms related to that specific group.

In this study, nine articles were included, involving three symptom assessment methods. The Chinese version of MSAS was used most frequently (n = 6), followed by MDASI (n = 3). However, none of these tools specifically assessed symptoms unique to leukemia patients. All included studies used quantitative research methods to identify symptom clusters and determined the symptom clusters of patients during chemotherapy through factor analysis. Although the same analytical method was used, differences in symptom assessment tools and measurement time points led to inconsistent types and numbers of symptom clusters identified. Changlan LIANG used the revised MDASI and found that there were four symptom clusters in leukemia patients during chemotherapy, namely the upper gastrointestinal symptom cluster, the nerve invasion symptom cluster, the psychological symptom cluster, and the energy deficiency symptom cluster.^[22]^ In contrast, Xixi YIN used MSAS and identified five symptom clusters, including the gastrointestinal symptom cluster, the psychological symptom cluster, and the energy deficiency symptom cluster.^[15]^ This indicates that different assessment tools can lead to changes in the types and numbers of symptom clusters. Meanwhile, MDASI, as a multidimensional assessment scale, evaluates not only the severity of symptoms but also their impact on six aspects of quality of life. However, in the included studies, one study used only the first part of the scale to determine the symptom clusters in leukemia patients during chemotherapy. What is more noteworthy is that one of the studies used a self-designed non-standardized questionnaire, and the symptom items were mostly selected based on the subjective experience of researchers, which may miss the key symptoms specific to leukemia during chemotherapy. At the same time, the results of self-designed questionnaire are difficult to compare with other studies, which seriously limits the reliability of its conclusions and the value of clinical promotion. In the future, it is necessary to develop standardized assessment tools for leukemia and verify their applicability through multicenter studies. At the same time, multi-method joint analysis was explored to clarify the mechanism of symptoms in the group, and to provide scientific basis for formulating individualized intervention strategies.

In summary, despite all studies targeting adult leukemia patients undergoing chemotherapy, significant differences still exist in the results of symptom cluster research. The presence of symptom clusters provides important clinical targets for developing effective treatment strategies. Clarifying the causal relationships and mechanisms among symptoms within clusters may better enable clinicians to identify and alleviate key symptoms that exacerbate other symptoms. Therefore, to optimize the management of symptom clusters during chemotherapy in leukemia patients, there is an urgent need to develop and standardize assessment tools. For multidimensional measurement tools, future research should expand sample sizes and validate across different populations and treatment stages to determine which dimensions are most suitable for symptom cluster research. Meanwhile, the current analytical methods in the field of symptom cluster research for leukemia patients are relatively limited. Future research should explore new analytical techniques, such as latent class analysis, machine learning, and network analysis, and compare multiple methods to identify the most clinically applicable approach.

## 5. Summary

This scoping review found that leukemia patients experience multiple symptom clusters during chemotherapy. However, research in this area is limited, and most existing studies are cross-sectional. Although these studies are helpful for timely discovering symptom clusters containing highly correlated symptoms, due to the lack of uniformity in symptom assessment and analysis methods and the large differences in symptom cluster composition, there are still certain limitations in finding intervention targets for implementing effective treatment strategies in clinical practice. In the future, it is still necessary to explore the assessment and analysis methods of symptom clusters in leukemia patients, verify the composition and characteristics of symptom clusters, and construct a precise symptom management plan based on the characteristics of symptom clusters in different chemotherapy stages. Future studies should apply machine learning to longitudinal datasets to predict dynamic symptom cluster trajectories, helping medical staff to find intervention targets at different stages and improve the efficiency and effectiveness of symptom management.

## Author contributions

**Conceptualization:** Shujia Liu, Lei Wang, Jiating Wang, Xia Yan.

**Data curation:** Shujia Liu, Lei Wang, Xia Yan.

**Formal analysis:** Shujia Liu.

**Methodology:** Shujia Liu, Lei Wang.

**Supervision:** Shujia Liu, Xia Yan.

**Validation:** Shujia Liu.

**Writing – original draft:** Shujia Liu, Lei Wang, Jiating Wang.

**Writing – review & editing:** Shujia Liu, Lei Wang, Jiating Wang, Xiaodong Xu, Kun Zhang, Ting Wang, Xia Yan.
